# Lambl’s Excrescence and Management of Recurrent Cerebrovascular Accident (CVA)

**DOI:** 10.7759/cureus.61681

**Published:** 2024-06-04

**Authors:** Amanda R Beering, Hanad Bashir, Santosh G Menon

**Affiliations:** 1 Internal Medicine, The Christ Hospital, Cincinnati, USA; 2 Cardiovascular Medicine, The Christ Hospital, Cincinnati, USA

**Keywords:** stroke, cva, valvular strand, cardio-embolic, lambl’s excrescence

## Abstract

Lambl’s excrescence is a rare valvular finding of uncertain pathologic significance. This case describes a previously healthy 42-year-old woman experiencing a sudden onset of word-finding difficulty. MRI of the brain demonstrated acute and chronic infarcts, prompting echocardiography, which revealed Lambl’s excrescence of the aortic valve.

## Introduction

Lambl’s excrescence is a rare potential cause of thromboembolic CVA and should be considered a significant contributing factor to CVA risk. It is important to obtain thorough cardiac imaging to evaluate the cardiac origin of CVA. TEE and cardiac MRI can help identify Lambl’s excrescence as a contributing factor in the workup of CVA of uncertain etiology. While no consensus currently exists for treatment, Lambl’s excrescence is commonly managed by pharmacologic and/or operative therapies, including antiplatelet and anticoagulant medications and consideration of valvular replacement.

## Case presentation

A 42-year-old female with a past medical history of psoriatic arthritis maintained on a stable dose of methotrexate and adalimumab developed sudden-onset word-finding difficulty and presented to the Emergency Department. She was otherwise in her normal state of health. She had no tobacco exposure, alcohol abuse, or illicit substance use. She had two previous normal pregnancies resulting in uncomplicated vaginal deliveries eight and ten years before the presentation and used an etonogestrel/ethinyl estradiol vaginal ring for birth control. She had no personal or family history of blood clots or strokes. She denied palpitations, chest pain, chest tightness, headaches, and dizziness. The physical exam was unremarkable, apart from intermittent, mild expressive aphasia.

A broad differential diagnosis was considered [1], including psychogenic aphasia, thrombotic CVA, atherosclerotic/arterioembolic CVA, including cardioembolic CVA, specifically PFO, paroxysmal atrial fibrillation, valvular vegetation, fibroelastoma, atrial myxoma, valvulopathy, and atrial septal defect.

**Figure 1:**
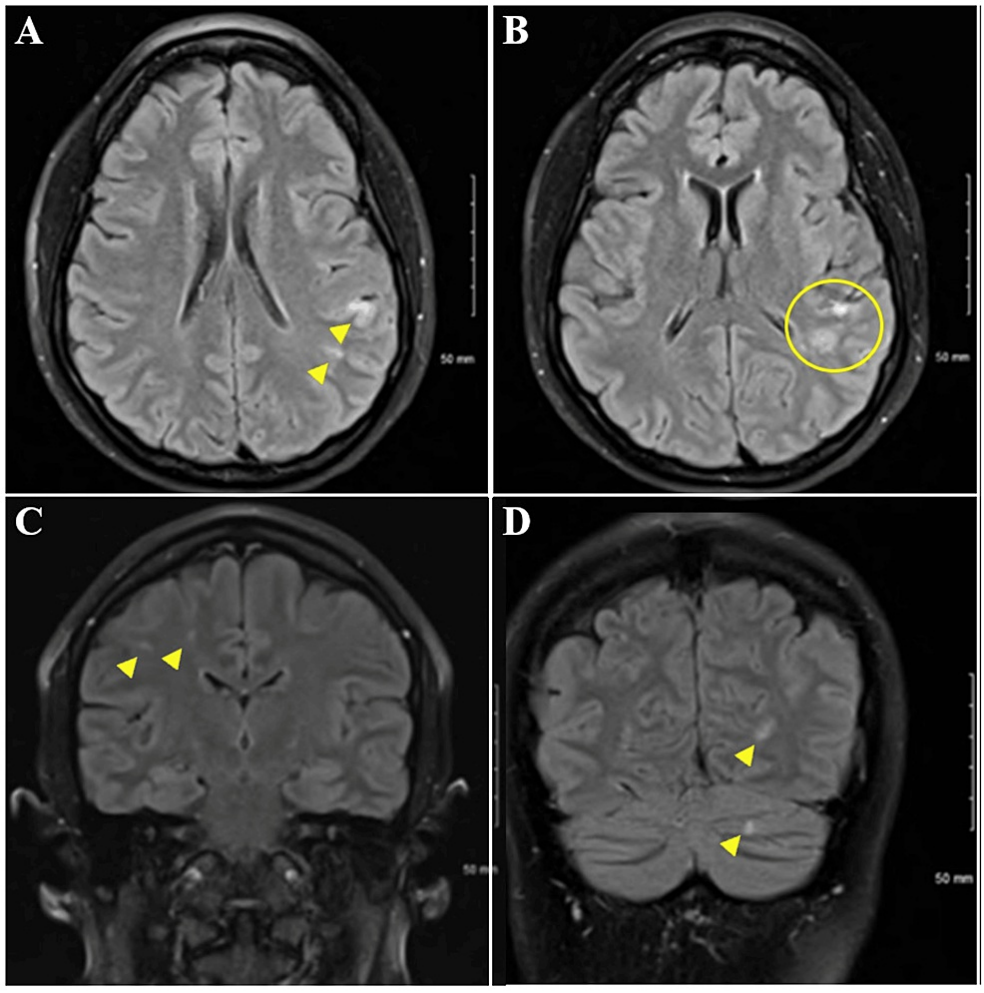
MRI brain, axial, and sagittal views Figure 1A: Acute infarction in the left parietal cortex and left parietotemporal lobe subcortical white matter (arrows) Figure 1B: Acute and subacute infarction in the left parietal cortex and left parietotemporal lobe subcortical white matter (circle) Figure 1C: Chronic small infarctions in the right parietal lobe Figure 1D: Chronic small infarction in the right posterior frontal subcortical white matter and left cerebellum.

The EKG showed a normal sinus rhythm. CT head and CTA head and neck were normal; however, the MRI brain demonstrated acute infarction in the left parietal cortex and left parietotemporal lobe subcortical white matter, as well as chronic small infarctions in the right parietal, right posterior frontal subcortical white matter, and left cerebellum (Figures 1A-1D).

**Figure 2:**
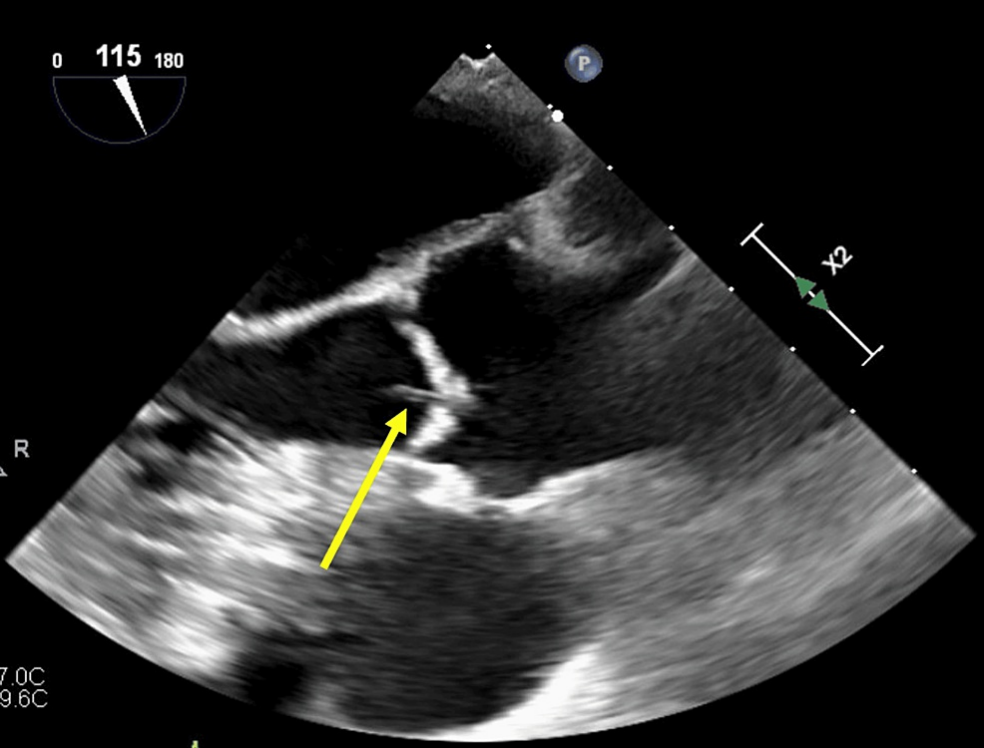
Transesophageal echocardiogram, midesophageal long-axis view Mobile filamentous structure attached to the aortic valve, consistent with Lambl’s excrescence (arrow).

**Figure 3:**
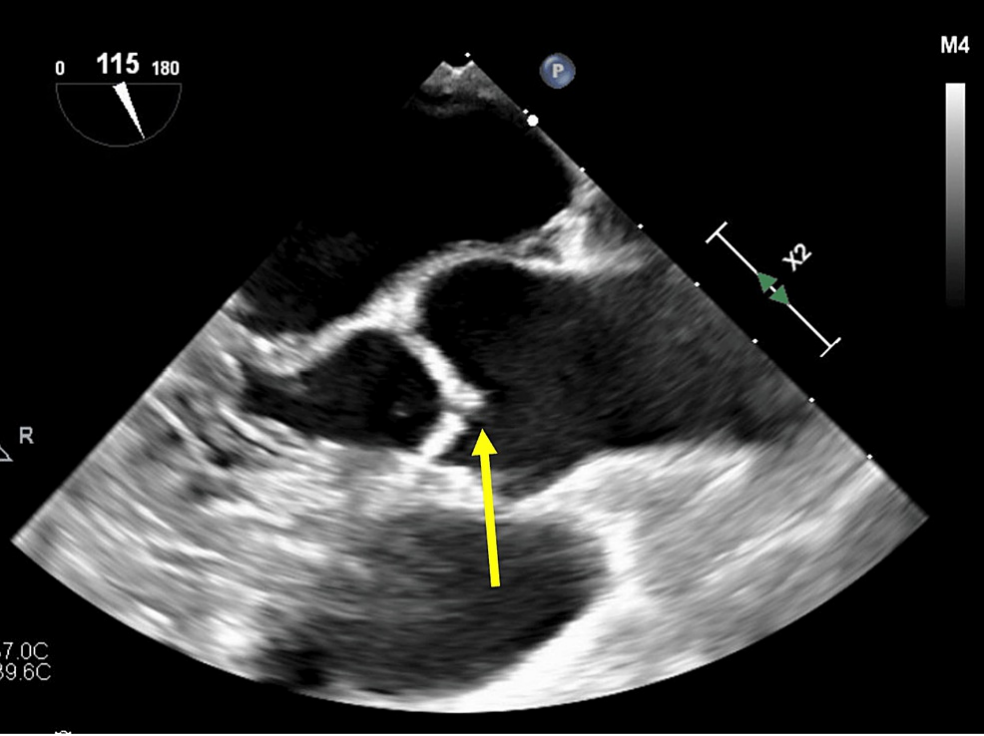
Transesophageal echocardiogram, midesophageal aortic valve, long-axis view Alternate image demonstrating different planes of the valvular strand (arrow), indicating the mobility of the filamentous structure attached to the aortic valve [2].

Given her young age and no known risk factors for stroke, she underwent TTE with a bubble study, which did not demonstrate evidence of PFO. TEE was performed for further consideration of possible cardiac sources of acute and chronic embolic infarcts and revealed a mobile filamentous structure attached to the aortic valve, consistent with Lambl’s excrescence (Figures 2, 3). A cardiac MRI excluded any intracardiac shunt.

Cardiothoracic surgery was consulted, and it was felt that operative intervention by open sternotomy was high-risk. The case was discussed by a multidisciplinary panel, with recommendations made to proceed with antiplatelet and anticoagulation therapies with apixaban and aspirin until definitive surgical management. The hematology/oncology workup was without concern for the hypercoagulable state. A recommendation was made for lifelong anticoagulation.

With approval from neurology, the patient was started on anticoagulation therapy with apixaban. She was discharged with a 30-day event monitor and plans to follow up with cardiology for ongoing management and cardiothoracic surgery for consideration of minimally invasive surgery to remove the growth. The 30-day event monitor did not reveal any arrhythmia.

The patient decided to seek a second opinion at an outside facility with regard to a possible surgical intervention. Surgeons at that facility recommended repeat hypercoagulability testing at six months and continued monitoring of apixaban and aspirin therapies.

## Discussion

The multifocal cortical and subcortical distribution, as well as the acute, subacute, and chronic infarcts demonstrated on MRI, are consistent with cardio-embolic etiology. Bubble study has been repeatedly negative for shunt, including transcranial Doppler bubble study performed during follow-up at an outside institution, and no further structural or hematologic abnormality has been identified, suggesting Lambl’s excrescence as the causative etiology of this patient’s strokes. No guidelines have been agreed upon with regard to the management of Lamb’s excrescence. Although a strong association has been demonstrated between Lambl’s excrescence and stroke, particularly among younger patients, it is unclear if the relationship between LE and CVA is causative or coincidental [3-5]. The remainder of this patient’s workup was unremarkable, suggesting Lambl’s excrescence as the causative etiology of this patient’s CVAs. In patients with LE and multiple episodes of CVA, such as in our case, tertiary prevention is warranted with either pharmacologic or operative management. Considerations guiding management decisions include comorbid risk factors, which were absent in this case. Given evidence of at least three separate incidences of CVA, careful consideration of excision is warranted to minimize the risk of recurrence [6,7]. She had no other indication or pathologic target for surgical intervention [8], and despite recurrent embolic events, she had not been previously treated with antiplatelet or anticoagulation and, therefore, had not failed therapy [9,10]. Given that she was not initially treated, it is impossible to know if she would have had three or more cardioembolic events despite therapy. Vitamin K antagonist therapy was considered; however, given a lack of evidence for superiority, it was not pursued as a treatment strategy due to the significant testing burden and potential interactions with other medications and foods [11,12]. The decision was made to manage conservatively with apixaban and aspirin dual therapy with continued close monitoring and defer escalation to procedural intervention unless and until the patient had an additional CVA despite pharmacologic therapy.

## Conclusions

This case highlights the importance of thorough cardiac imaging when evaluating a patient presenting with CVA to identify cardioembolic pathologies. Recognition of Lambl's excrescence as a potential cause of thromboembolic CVA is vital for CVA risk assessment, and thorough cardiac imaging, including TEE and CMR, should be considered in patients presenting with CVA of uncertain origin. This allows for consideration of additional therapeutic options, both pharmacologic and operative, as well as providing information pertinent to consideration of the risks and benefits of each treatment modality. Consideration should be given to lifelong treatment with both antiplatelet and anticoagulant therapy in patients with Lambl's excrescence who do not undergo procedural intervention, as a potential mechanism of CVA has not been eliminated.
